# Onset Mechanisms and Prognosis of Neurally Mediated Syncope

**DOI:** 10.3390/reports6040056

**Published:** 2023-11-30

**Authors:** Tomoyoshi Komiyama, Kengo Ayabe, Misaki Hasegawa, Marie Yoshikawa, Susumu Sakama, Kyong-Hee Lee, Atsuhiko Yagishita, Mari Amino, Eiichiro Nagata, Yuji Ikari, Koichiro Yoshioka, Hiroyuki Kobayashi

**Affiliations:** 1 Department of Clinical Pharmacology, Tokai University School of Medicine, Isehara 259-1193, Kanagawa, Japan; hkobayas@is.icc.u-tokai.ac.jp; 2 Cardiovascular Center, Miyazaki Medical Association Hospital, Miyazaki 880-2102, Miyazaki, Japan; 3 Department of Cardiology, Tokai University School of Medicine, Isehara 259-1193, Kanagawa, Japan; hhshmm.0726@gmail.com (M.H.); ma-ri-e0604@outlook.jp (M.Y.); afrosick925_4amm1408@yahoo.co.jp (S.S.); seraph418@gmail.com (K.-H.L.); ayagishita@tsc.u-tokai.ac.jp (A.Y.); mariam@is.icc.u-tokai.ac.jp (M.A.); ikari@is.icc.u-tokai.ac.jp (Y.I.); ko1yoshioka@gmail.com (K.Y.); 4 Department of Neurology, Tokai University School of Medicine, Isehara 259-1193, Kanagawa, Japan; enagata@is.icc.u-tokai.ac.jp

**Keywords:** neurally mediated syncope, systolic blood pressure, adenylate cyclase, head-up tilt, Giα-protein-coupled receptors

## Abstract

Neurally mediated syncope (NMS) is associated with a sudden loss of consciousness. However, the diagnostic tools and measures for prognosis management are limited. To overcome these limitations, the differences in the binding energies of Giα-protein-coupled receptors to the Glu9 and Glu12 residues on the *α2B-AR* gene were elucidated through the analysis of *α2B-AR* gene polymorphism. The suppression of the activity of adenylate cyclase (AC), which is involved in vasoconstriction, may be related to the onset of NMS. The head-up tilt (HUT) test results indicated differences in systolic blood pressure (SBP) and AC activity between patients with vasodepressor (VT)-NMS and healthy volunteers. Patients with VT-NMS had increased AC activity and decreased SBP. Conversely, in healthy volunteers, no changes in AC activity or SBP were found. These findings suggest that a high SBP and elevated AC activity at rest are likely to cause syncope. A high incidence of cardiovascular events is found in patients with negative HUT test results, highlighting the importance of investigating the cause of syncope in cases where the HUT test results are negative. Overall, our results may provide a means of assessing the risk of NMS development within healthy populations and underscore the importance of subsequent treatments for NMS.

## 1. Introduction

Syncope is a temporary loss of consciousness in which the person is unable to maintain an upright position, followed by a spontaneous and complete recovery of consciousness [1,2]. It commonly results from a transient hypoperfusion of the entire brain, which can be triggered by various diseases [1,3,4]. Neurally mediated syncope (NMS) occurs frequently and interferes with daily life activities [5,6]. However, patients typically recover by the time of examination; therefore, the underlying mechanisms of onset are unclear and the treatment methods available are limited [7,8,9,10,11]. NMS itself is not fatal; however, its sudden onset can lead to head injury from falls and traffic accidents due to fainting, potentially causing harm to both the patient and those in close proximity [7,12]. Numerous laboratory tests and imaging models are available for identifying the causes of syncope. However, because of its diverse etiologies, the clinical approach may not be able to sufficiently clarify the underlying mechanisms of NMS. A more thorough approach would be from a molecular biology standpoint, as the genome is indispensable for understanding this condition. Consequently, researchers investigating syncope encompass a range of medical specialists, including internists, cardiologists, neurologists, and pharmacologists. The frequency of unexplained medical conditions can vary in clinical studies and investigations; however, NMS is the most common type of syncope, having been recorded in 17–37% of the Japanese population [7,13,14,15,16,17]. 

In one of our previous studies, the alpha-2B adrenergic receptor (α2B-AR; Glu301–303) gene was analyzed in patients with NMS [18]. The activation of α2B-AR induces the suppression of adenylate cyclase (AC) by the Gi protein, leading to the constriction of blood vessels. Conversely, β2 receptors bind to and activate Gs proteins, resulting in the relaxation of blood vessels. Therefore, based on genetic analyses, the onset and cause of NMS were investigated by measuring AC activity in each patient, both at rest and during head-up tilt (HUT test) [18,19]. AC strongly affects the contractile function of vascular smooth muscles by producing cyclic adenosine mono-phosphate (cAMP), and this process is associated with syncope [18,20,21,22,23]. Furthermore, the findings of our HUT study indicated that patients with negative HUT test results experienced more cardiac events that led to syncope than those with positive ones [24]. Therefore, changes in AC activity in the body during stress and α2B-AR gene polymorphisms may be useful for the assessment of the risk of syncope attacks in patients with NMS. In this review, the onset mechanisms, prevention, and prognosis of NMS from both clinical and basic perspectives were examined based on the findings of our previous studies [18,19,24,25].

## 2. Mechanism Underlying the Molecular Interactions between α2B-AR Gene Polymorphisms and Gi Protein in NMS

The first interest in the AC activity of patients with NMS from a research perspective was demonstrated by Small et al. They reported that AC activity is suppressed in cells with a Glu 12 repeat, as observed in experimental Chinese hamster ovary cells [26].

In a previous study, we investigated the factors contributing to NMS by analyzing the interaction mechanism of the polymorphisms within the two subtypes of the beta-2 adrenergic receptor (*ADR-β2*) gene with the G-protein-coupled receptors. To achieve this, we conducted pathophysiological and clinical epidemiological surveys. We focused on the three types of glutamate repeats (E: p.Glu301–303) found in the *α2B-AR* gene: Glu12/12 homozygous, Glu9/9 homozygous, and Glu9/12 heterozygous [18] (Table 1). In our previous studies, nine patients with NMS had heterozygous Glu repeats, which were found to influence cAMP activity within the body. This phenomenon potentially impacts vasoconstriction and contributes to the recurrence of NMS episodes. The result obtained is based on α2B-AR activation, which causes the Gi protein to suppress the activity of AC, resulting in the constriction of blood vessels. Conversely, through the action of β2 receptors, α2B-AR binds to and activates Gs proteins, resulting in the relaxation of blood vessels [27,28,29,30]. Furthermore, when cAMP activates protein kinase A, the calcium ion channels open, which promotes calcium uptake and affects the contractile force of the smooth muscle [20,21,22,31,32,33,34]. In our previous study, we examined the Glu301–303 glutamate repeat polymorphism site in the *α2B-AR* gene [30], and the differences in the signaling times of Glu12/12, Glu12/9, and Glu9/9 were evaluated. When measuring the differences in the signal transduction time of the Gi (α) subunits (GPCRs), we observed that the binding energy between the Gi (α) subunits and Glu9 (−77.94) was higher than that between the Gi (α) subunits and Glu12 (−68.40) [19] (Table 2). These findings imply that the receptor included in the Glu12 repeats can be released quickly from the α subunit and rapidly influence AC signaling. Additionally, the changes in AC activity can immensely affect the contractile function of vascular smooth muscle and be strongly involved in the onset of syncope. Changes in the concentration of norepinephrine and epinephrine associated with the different postural changes made during the HUT test have been reported [35,36]. Previous studies have reported higher catecholamine levels in patients at rest, compared with the levels observed just before an episode of syncope [35,36,37,38]. The cAMP level was also higher prior to the fainting episodes [39,40,41,42]. The current method used for diagnosing NMS in Japan is the HUT technique, which is effective for diagnosing high-risk cases that only have a single syncope or organic heart disease event. This review may identify the potential variants related to NMS diagnosis through catecholamine receptor gene analysis and facilitate the understanding of the association between the α2B-AR gene and NMS.

## 3. Relationship between AC Activity and the Onset of NMS

**Figure 1:**
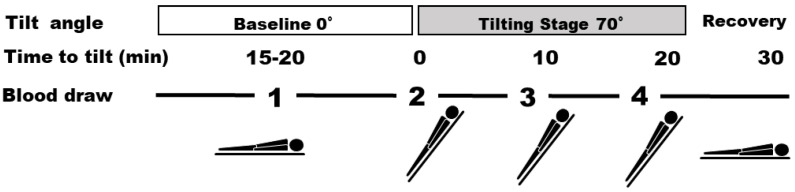
Head-up tilt (HUT) protocol and the four time points for blood collection.

The AC levels in the lymphocytes of patients with NMS were examined in one of our previous studies. To the best of our knowledge, this was the first study to report the relationship between AC activity and NMS [18]. The study included 29 volunteers: 12 healthy volunteers and 17 patients with NMS. The changes in AC activity that occurred during syncope, according to the HUT test results, were investigated. We collected 8–10 mL of blood from the volunteers four times during the HUT test (Figure 1), followed by the measurement of AC activity. The test reagent was added to the lymphocytes (10,000) and incubated for 30 min at approximately 25 °C.

Adrenaline (AD) and isoproterenol (IP) were added to the lymphocytes to determine the standard AC activity value. We found that patients could be classified into two NMS categories based on the differences in AC activity: vasodepressor type (VT) or mixed type (MT). The VT patients had substantially higher AC activity than healthy volunteers at all the four points of the HUT test (Table 3 and Table 4). However, the AC activity in MT patients did not significantly differ from that of healthy volunteers at the four points (Table 5 and Table 6). The patients with NMS and healthy volunteers showed substantial differences in AC activity at rest and during the HUT test. The two types (VT and MT) of patients had different activity levels. Notably, we could diagnose these patients with NMS. Therefore, these data suggest that healthy individuals at high risk of developing NMS can be diagnosed. Furthermore, no change in AC activity was observed among patients with different HUT test results or among different time points (separated by 11, 5, or 2 months) in the three healthy volunteers. The determination of the level of AC activity may allow for the early diagnosis of at-risk patients. Even in environments where diagnosing VT and MT patients is difficult, AC activity can be a useful tool for diagnosing NMS.

## 4. Systolic Blood Pressure (SBP) and AC Activity in Japanese Patients with VT-NMS

**Figure 2:**
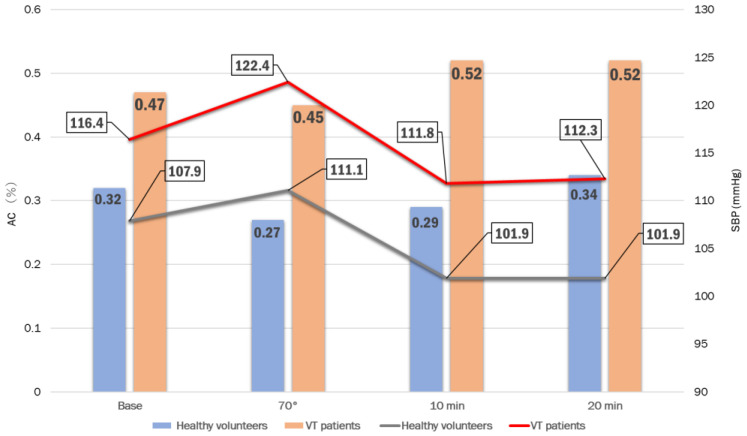
Changes in systolic blood pressure (SBP) and adenylate cyclase (AC) activity in patients with vasodepressor-type (VT) neurally mediated syncope (NMS) and healthy volunteers during the head-up tilt (HUT) test [25].

In another study, VT, which was the most frequent type among the patients with NMS in our hospital (Tokai University School of Medicine, Kanagawa, Japan) between January 2016 and May 2020, was investigated [25]. We evaluated a total of 124 patients with suspected NMS with an average age of 49.3 ± 21.6 years. The transthoracic echocardiography results showed an average left ventricular ejection fraction of 67.8 ± 8.8%. The average age of the VT patients was 51.4 ± 25.3 years (male: 54.7 ± 23.2 years; female: 44.9 ± 23.6 years), and that of the negative patients was 54.5 ± 19.0 years (male: 57.4 ± 19.3 years; female: 46.7 ± 16.8 years), indicating that no statistically significant sex- or age-related differences were observed. In addition, no changes in autonomic variability during electrocardiography were observed in the VT patients. However, 19 patients with VT-NMS and 11 with negative HUT test results underwent high-resolution ambulatory Holter electrocardiography (ECG). The left ventricular ejection fractions measured using transthoracic echocardiography were 69.9 ± 8.8% and 65.6 ± 11.0% for VT-NMS and negative patients, respectively. The number of premature ventricular contractions was significantly higher in the negative HUT group than in the VT-NMS group (611.7 ± 1061.9 vs. 6.6 ± 10.7; *p* = 0.018). In contrast, during the HUT study, the AC activity in patients with VT-NMS was the highest (0.52%) after 10 min (Table 7). The systolic blood pressure (SBP) of the VT patients decreased 5 min after standing and became the lowest (111.8 mmHg) after 10 min (Table 8a). Their pulse rate increased after 10 min (Figure 2). The patients with VT-NMS showed a higher blood pressure (BP), pulse rate, and AC activity during the HUT test than the healthy volunteers. Furthermore, the patients with VT-NMS showed a substantially higher BP, pulse rate, and AC activity than the healthy volunteers; this pattern was also observed for SBP and AC activity in the volunteers at rest. In patients with syncope, standing for >10 min may increase the risk of VT-NMS. As a result, high SBP and AC activity at rest may cause fainting in patients with VT-NMS. These findings may help to identify individuals at high risk of developing NMS in a healthy population.

## 5. Clinical Significance of the HUT Test for Improving the Prognosis of Patients with Possible NMS

One of our previous studies investigated the clinical significance of performing the HUT test on patients with syncope. The charts of 101 patients who underwent the test at Tokai University Hospital between January 2016 and March 2019 were reviewed [24]. To rule out the possible cardiac and neurological causes of syncope, blood tests, ECG, echocardiography, and electroencephalography, if indicated, were performed for all patients prior to the HUT test. NMS was the most likely cause of syncope at the time of the HUT test. In total, 72 patients (69.2%) tested positive in the HUT test, indicating that they had syncope. The patients were followed up for 886.1 ± 457.7 days (interquartile range: 518–1293 days). The rate of syncope recurrence was 16.9%; however, no significant differences were observed between the two groups (positive vs. negative HUT test: 13.8% vs. 18.1%, *p* = 0.772). Four of the 29 (13.9%) negative HUT patients and two of the 72 (2.8%) positive patients experienced cardiac events (*p* = 0.019) [24]. Any negative HUT test results obtained could be due to unexpected clinical events within a few years of the test. The NMS diagnosis most likely to be correct is that made at the time of the HUT test; however, negative results may require a reconsideration of the initial diagnosis based on the clinical information available. Close outpatient follow-ups were required for those who tested negative. The HUT test is not a standard approach to diagnose NMS; therefore, the results should be interpreted with caution. The sensitivity and specificity were around 57.5–87% and 70–100%, respectively [43]. A positive NMS test result implies that the cause of syncope is likely NMS. In particular, ruling out other etiologies before performing a HUT test is essential because the sensitivity and specificity of this test are not 100%. However, if the HUT test results are negative, physicians must interpret the results with caution and should not conclude that the HUT test is negative due to its inaccurate sensitivity rate. Other etiologies, such as cardiac and neurological causes, were already ruled out prior to the HUT test; however, the possible causes of syncope other than NMS needed to be investigated more actively. Implantable cardiac monitors (ICMs) play an important role in diagnosing the cause of unexplained syncope. The findings indicate that the patients with negative HUT test results had higher rates of cardiac events that led to syncope than those with positive HUT test results. Therefore, negative HUT test results may indicate the need for ICM placement in patients with syncope.

## 6. Discussion

The HUT test is a widely used approach for diagnosing NMS. However, it requires overnight hospitalization, and the number of facilities where HUT tests can be performed is limited. Moreover, the burden inflicted on patients is significant. Therefore, alternative, less intrusive diagnostic methods should be explored. Heart rate variability (HRV) is a non-invasive method used for assessing the regulation of the autonomic nervous system [44,45,46]. This method can be assessed using a 24 h Holter or resting ECG. In one of our previous studies, we used a 24 h Holter ECG to diagnose arrhythmias [47,48,49]. The autonomic nervous system is involved in syncope [7]; hence, the evaluation is performed in the time and frequency domains. Therefore, HRV can be used as a diagnostic indicator of NMS. Multiple HRV studies have shown that patients with NMS exhibit increased autonomic nervous system activity in their daily lives. However, Sneddon et al. reported no significant differences between patients with NMS and healthy volunteers [50]. Lazzeri et al. reported a low standard deviation of the 5 min mean NN interval for each syncopal episode [51]. A high-resolution Holter ECG can be used to evaluate the autonomic nerves through HRV analysis [44,45,46,47,48,49,50,51].

Changes in high-resolution Holter ECG, AC activity during the HUT test, blood pressure (BP), and heart rate (HR) were analyzed in patients with VT-NMS, and the hemodynamic changes were reviewed based on the findings of our previous studies. By analyzing the changes in the pathological condition of patients with NMS during stress, we investigated whether the BP, HR, and AC activity were effective predictors of NMS syncope recurrence.

NMS is associated with a good prognosis; therefore, it is often underestimated in daily clinical practice. Outpatient follow-ups were discontinued at the discretion of the patient or the physician [52]. Limited treatment methods, such as pharmacotherapy and orthostatic training, also contributed to insufficient outpatient follow-ups [53]. Therefore, only a few studies have been conducted on the reoccurrence of syncope after a HUT test. In one of our previous studies [24], with a follow-up of 886.1 ± 457.7 days, the recurrent syncope rate was 16.9%. A high syncope recurrence rate of 32.5% was reported in older patients [52]. The median age of the patients in our data [24] was 49.6 ± 21.0 years; therefore, the recurrence rate was lower than that previously reported [54]. The rate of recurrence of syncope also differed between the positive and negative patient groups. However, our study did not consider the syncope rates in patients who did not undergo an HUT test [24]. All patients were instructed to undergo orthostatic training at home after discharge. Conflicting results have been reported regarding the effectiveness of orthostatic training; however, the high adherence rate may have contributed to the low rate of recurrent syncope observed in our patients [52,55]. Further research is required to determine whether adherence to orthostatic training is associated with recurrent syncope. Furthermore, we found that the patients with negative HUT test results were more likely to develop unexpected heart disease at a statistically significant (*p* = 0.019) frequency than those with positive ones. Therefore, more in-depth outpatient monitoring, such as performing 24 h Holter ECG and blood tests, is recommended for the investigation of the etiologies of syncope. European and American guidelines agree that the use of an implantable loop recorder for syncope should be determined by considering the patient characteristics and syncope frequency and by pre-testing the probability of an arrhythmic cause of syncope [56,57]. Atrial fibrillation (AF)-associated arrhythmias are a common cause of fatigue in the older population. Older individuals with a negative HUT test should have an implantable loop recorder inserted to monitor any intracardiac conduction disturbances and AF [58].

Our previous study was a single-center retrospective study with a small number of patients [25], which raised a selection bias problem. NMS reportedly occurs more frequently in women than in men [59]; however, we observed that it occurs more frequently in men. Furthermore, studies including more patients are required to validate these results. The differences in AC activity, BP, and HR variations between patients with VT-NMS and MT patients were further confirmed during the HUT test. Furthermore, in the HUT test, the patients’ SBP decreased after 10 min of standing, affecting the hemodynamics before syncope. An increased AC activity is believed to cause vasodilation and syncope in patients with VT-NMS. These results show that patients with VT-NMS are likely to have syncope attributable to their high resting SBP and AC activity.

These results further suggest that patients with syncope characterized by high resting AC activity and SBP are more likely to be NMS-positive. This finding supports previous reports and may be the basis of diagnosis and treatment at facilities where HUT tests cannot be performed. Patients with VT-NMS have a high resting SBP and AC activity; hence, lifestyle guidance is necessary to prevent any increase in AC activity. In patients with syncope, standing for > 10 min increases the risk of developing VT-NMS because AC synthesizes cAMP from ATP [32,33,34]. The action of α2B-ARs causes the Gi protein α subunits to suppress AC activity, resulting in vasoconstriction [26,31,60,61,62]. Additionally, β2-ARs emit signals that relax the blood vessels by binding to Gs proteins [61,63]. Furthermore, the cAMP activation of protein kinase A opens the calcium ion channels, promoting calcium uptake and modulating smooth muscle contractility [20,21,22,23,64,65,66,67]. Consequently, an increase in AC activity may lower the SBP. In patients with VT-NMS, the AC activity increased before NMS and the SBP decreased. However, no changes in AC activity or SBP were observed in healthy volunteers. Patients with syncope characterized by high AC activity and resting SBP are likely to have positive HUT test results and VT. Instructing patients with suspected VT-NMS to avoid stressful situations, such as dehydration, standing for more than 10 min, high temperatures, or crowded environments, may help to prevent syncope. Several studies have suggested a relationship between caffeine intake and increased AC activity [68,69,70]. Patients with NMS who have higher AC activity and SBP than those at rest are advised to avoid caffeine. No studies on the preventive effect of lifestyle guidance on fainting exist; however, reducing the intake of caffeine can have a substantial effect on suppressing recurrent fainting.

This study has some limitations. The results were obtained from a small number of patients with NMS and healthy volunteers. We performed both biochemical and HUT tests in each patient with syncope in a single day to identify individuals with NMS. We had to proceed with caution during the HUT test because it exerts stress on both patients and volunteers. In fact, approximately 20% of the volunteers experienced NMS during the HUT test. The symptoms of NMS occur in stressful situations. Moreover, healthy individuals with poor physical performance in the HUT test were excluded from data analysis.

## 7. Conclusions

The findings of this study reconfirm the HUT test as an appropriate approach for diagnosing NMS. Furthermore, our results revealed that patients with VT-NMS had increased AC activity and a decreased SBP. Conversely, in healthy volunteers, no changes in AC activity or SBP were found. In the future, further studies on new patients with NMS and healthy volunteers are essential to increase the statistical reliability of the data. Our goal is to leverage these research findings for the development of novel diagnostics, encompassing the assessment of the Glu9 or Glu12 repeat frequency in the *α2B-AR* gene, the difference in AC activity, and treatment methods for NMS in seemingly healthy patients. The results obtained herein may make a substantial contribution to the fields of social medicine, cardiology, and neurology.

## Figures and Tables

**Table 1:** Glu repeat numbers in the patients with neurally mediated syncope (NMS) and healthy volunteers [18].

Allele Type	Total	Glu12/12	Glu12/9	Glu9/9
NMS	9	1	8	0
Healthy volunteers	11	3	5	3

Glu, glutamate; NMS, neurally mediated syncope.

**Table 2:** Binding energy between the Gi protein α-subunit and the Glu9 and Glu12 residues on the *α2B-AR* gene [18].

	Glu9	Glu12
Binding energy of the Gi protein α-subunit ^a^	−77.94	−68.40

^a^ Per residue binding energy, as determined using in silico analysis.

**Table 3:** Adenylate cyclase (AC) activity in patients with vasodepressor-type (VT) neurally mediated syncope (NMS) and healthy volunteers during the head-up tilt (HUT) test using isoproterenol (IP) [19].

Patients with NMS (VT) *n* = 11	Average (%)	SD (%)	Healthy Volunteers *n* = 12	Average (%)	SD (%)	*p* Value
**Base**			**Base**			
IP 50 µM	0.560415	0.152698	IP 50 µM	0.404779	0.154299	0.01212
IP 5 µM	0.470369	0.170573	IP 5 µM	0.330279	0.15716	0.027111
**70°**			**70°**			
IP 50 µM	0.522241	0.148235	IP 50 µM	0.378662	0.156597	0.017344
IP 5 µM	0.435144	0.156784	IP 5 µM	0.304623	0.156206	0.029483
**10 min**			**10 min**			
IP 50 µM	0.586415	0.180438	IP 50 µM	0.396493	0.132411	0.00519
IP 5 µM	0.508118	0.204569	IP 5 µM	0.312684	0.134018	0.007836
**20 min**			**20 min**			
IP 50 µM	0.615201	0.187159	IP 50 µM	0.423144	0.119647	0.005006
IP 5 µM	0.542883	0.206041	IP 5 µM	0.331232	0.127212	0.004782

AC, adenylate cyclase; IP, isoproterenol; NMS, neurally mediated syncope; VT, vasodepressor type; HUT, head-up tilt; SD, standard deviation.

**Table 4:** Adenylate cyclase (AC) activity in patients with vasodepressor-type (VT) neurally mediated syncope (NMS) and healthy volunteers during the head-up tilt (HUT) test using adrenaline (AD) [19].

Patients with NMS (VT) *n* = 11	Average (%)	SD (%)	Healthy Volunteers *n* = 12	Average (%)	SD (%)	*p* Value
**Base**			**Base**			
AD 100 µM	0.653258	0.107929	AD 100 µM	0.541178	0.097983	0.008511
AD 10 µM	0.471868	0.168761	AD 10 µM	0.326923	0.149877	0.021068
**70°**			**70°**			
AD 100 µM	0.618596	0.104676	AD 100 µM	0.50587	0.118856	0.012396
AD 10 µM	0.430308	0.15054	AD 10 µM	0.287633	0.143551	0.015306
**10 min**			**10 min**			
AD 100 µM	0.658657	0.126682	AD 100 µM	0.496051	0.100559	0.00153
AD 10 µM	0.504208	0.186068	AD 10 µM	0.296339	0.112927	0.002731
**20 min**			**20 min**			
AD 100 µM	0.669466	0.184746	AD 100 µM	0.514371	0.089895	0.012058
AD 10 µM	0.544377	0.20292	AD 10 µM	0.334034	0.127794	0.004623

AC, adenylate cyclase; AD, adrenalin; NMS, neurally mediated syncope; VT: vasodepressor type; HUT, head-up tilt; SD, standard deviation.

**Table 5:** Adenylate cyclase (AC) activity in patients with mixed-type (MT) neurally mediated syncope (NMS) and healthy volunteers during the head-up tilt (HUT) test using isoproterenol (IP) [19].

Patients with NMS (MT) *n* = 6	Average (%)	SD (%)	Healthy Volunteers *n* = 12	Average (%)	SD (%)	*p* Value
**Base**			**Base**			
IP 50 µM	0.311706	0.076293	IP 50 µM	0.404779	0.154299	0.053075
IP 5 µM	0.24143	0.069333	IP 5 µM	0.330279	0.15716	0.058083
**70°**			**70°**			
IP 50 µM	0.297053	0.091338	IP 50 µM	0.378662	0.156597	0.091763
IP 5 µM	0.217388	0.087596	IP 5 µM	0.304623	0.156206	0.074785
**10 min**			**10 min**			
IP 50 µM	0.444013	0.163987	IP 50 µM	0.396493	0.132411	0.276989
IP 5 µM	0.371646	0.199079	IP 5 µM	0.312684	0.134018	0.266197
**20 min**			**20 min**			
IP 50 µM	0.491652	0.167788	IP 50 µM	0.423144	0.119647	0.459322
IP 5 µM	0.408247	0.139467	IP 5 µM	0.331232	0.127212	0.421843

AC, adenylate cyclase; IP, isoproterenol; NMS, neurally mediated syncope; MT, mixed type; HUT, head-up tilt; SD, standard deviation.

**Table 6:** Adenylate cyclase (AC) activity in patients with mixed-type (MT) neurally mediated syncope (NMS) and healthy volunteers during the head-up tilt (HUT) test using adrenaline (AD) [19].

Patients with NMS (MT) *n* = 6	Average (%)	SD (%)	Healthy Volunteers *n* = 12	Average (%)	SD (%)	*p* Value
**Base**			**Base**			
AD 100 µM	0.493567	0.049775	AD 100 µM	0.541178	0.097983	0.095287
AD 10 µM	0.251196	0.048196	AD 10 µM	0.326923	0.149877	0.066226
**70°**			**70°**			
AD 100 µM	0.453367	0.04789	AD 100 µM	0.50587	0.118856	0.101355
AD 10 µM	0.22152	0.068808	AD 10 µM	0.287633	0.143551	0.102607
**10 min**			**10 min**			
AD 100 µM	0.554954	0.1411	AD 100 µM	0.496051	0.100559	0.194535
AD 10 µM	0.370648	0.18302	AD 10 µM	0.296339	0.112927	0.196221
**20 min**			**20 min**			
AD 100 µM	0.583728	0.10373	AD 100 µM	0.514371	0.089895	0.156635
AD 10 µM	0.409066	0.134564	AD 10 µM	0.334034	0.127794	0.349466

AC, adenylate cyclase; AD, adrenaline; NMS, neurally mediated syncope; MT, mixed type; HUT, head-up tilt; SD, standard deviation.

**Table 7:** Adenylate cyclase (AC) activity in patients with vasodepressor-type (VT) neurally mediated syncope (NMS) and healthy volunteers (Unit: %) [25].

	Base	70°	10 min	20 min
**Healthy volunteers** ***n* = 15**	0.32 ± 0.14	0.27 ± 0.14	0.29 ± 0.10	0.34 ± 0.12
**patients with VT-NMS** ***n* = 27**	0.47 ± 0.18	0.45 ± 0.19	0.52 ± 0.21	0.52 ± 0.21
***p*** **value**	0.0051	0.0020	0.0002	0.0031

AC, adenylate cyclase; NMS, neurally mediated syncope; VT, vasodepressor type.

**Table 8:** Blood pressure (BP) and heart rate (HR) of patients with vasodepressor-type (VT) neurally mediated syncope (NMS) and healthy volunteers (unit: mmHg) [25].

**a. SBP**	**Base**	**70°**	**3 min**	**5 min**	**10 min**	**15 min**	**20 min**
**Patients with VT-NMS**	116.4 ± 14.1	122.4 ± 14.0	123.9 ± 18.4	118.1 ± 16.6	111.8 ± 19.8	111.3 ± 21.4	112.3 ± 16.9
**Healthy volunteers**	107.9 ± 8.0	111.1 ± 15.5	108.8 ± 9.1	107.1 ± 12.6	101.9 ± 10.0	104.4 ± 10.7	101.9 ± 9.1
***p* value**	0.036	0.020	0.019	0.032	0.090	0.264	0.037
**b. DBP**	**Base**	**70°**	**3 min**	**5 min**	**10 min**	**15 min**	**20 min**
**Patients with VT-NMS**	74.0 ± 12.0	80.6 ± 11.9	81.0 ± 11.6	78.6 ± 11.8	76.5 ± 13.5	74.3 ± 14.4	76.8 ± 15.1
**Healthy volunteers**	66.2 ± 8.8	79.7 ± 8.5	78.1 ± 7.8	74.5 ± 8.1	72.4 ± 10.6	73.6 ± 10.7	74.7 ± 8.7
***p* value**	0.032	0.815	0.472	0.248	0.335	0.865	0.640
**c. HR**	**Base**	**70°**	**3 min**	**5 min**	**10 min**	**15 min**	**20 min**
**Patients with VT-NMS**	66.7 ± 14.1	78.5 ± 16.8	81.0 ± 15.8	82.1 ± 17.0	82.1 ± 18.1	84.3 ± 19.6	86.9 ± 21.6
**Healthy volunteers**	65.7 ± 8.9	74.3 ± 7.6	74.7 ± 6.8	77.1 ± 7.4	75.6 ± 7.4	78.3 ± 8.5	79.7 ± 8.0
***p* value**	0.818	0.360	0.233	0.282	0.212	0.282	0.232

NMS, neurally mediated syncope; VT, vasodepressor type; BP, blood pressure; SBP, systolic blood pressure; DBP, diastolic blood pressure; HR, heart rate.

## Data Availability

Not applicable.
